# Metastatic Pancreatic Acinar Cell Carcinoma: An Unlikely Culprit

**DOI:** 10.7759/cureus.38288

**Published:** 2023-04-29

**Authors:** Alena Bashinskaya, Jay Kammerman, David Butson, Patricia Moody

**Affiliations:** 1 Osteopathic Medicine, Dr. Kiran C. Patel College of Osteopathic Medicine, Nova Southeastern University, Clearwater, USA; 2 Dermatology, HCA Florida Brandon Hospital, Brandon, USA; 3 Dermatopathology, KorPath, Tampa, USA

**Keywords:** metastatic pancreatic mass, scalp lesion, acinar cell carcinoma, cutaneous metastasis, pancreatic malignancy

## Abstract

Although acinar cells comprise a large volume of the pancreas, they rarely transform into malignant neoplasms. Once they arise, they rapidly metastasize via hematogenous spread to other organs such as the brain, liver, lung, and skeletal system. Cutaneous involvement, however, is rarely seen in all patients with primary pancreatic neoplasms. The most frequently reported site of cutaneous manifestations is the umbilicus, with the other sites including the trunk, lower extremities, head, and neck. Here, we report a case of metastatic pancreatic acinar cell carcinoma with cutaneous involvement of the patient's scalp.

## Introduction

Cutaneous metastasis arising from pancreatic cancer is a rare finding, with only a few cases documented in the literature. Pancreatic cancer carries a high malignancy potential and is the fourth leading cause of all cancer-related deaths in the United States [1]. Environmental exposure, epidemiologic diseases, blood types, and certain genetic markers, such as KRAS, p16, and p53, play an integral role in pancreatic carcinogenesis [2]. Pancreatic carcinomas typically arise in white males, with an average age of onset around the sixth decade of life [2]. Malignant tumors generally develop on the outer surface of the pancreas and exhibit a high proliferative rate, thus leading to rapid metastasis to other visceral organs [3]. While ductal adenocarcinoma represents the most common type of malignancy of the pancreas, pancreatic acinar cell carcinoma (PACC) comprises less than 2% of all pancreatic cancers and results in the overproduction of pancreatic enzymes by the neoplastic cells [4]. Although acinar cells comprise a large volume of the pancreas, they rarely transform into tumors [4]. Cutaneous involvement is so rare that it can only be found in less than 1% of all patients with pancreatic malignancies [5]. The most frequently reported cutaneous lesion is Sister Mary Joseph’s umbilical nodule, while other sites include the lower extremities, trunk, and scalp [6]. Lesions appear as tender nodules or ulcerative plaques with associated erythema and swelling [6]. To date, there have been fewer than 30 reported cases of cutaneous pancreatic metastases found in the head and neck region [5,6]. Herein, we report the novel occurrence of cutaneous involvement by PACC on a patient’s scalp.

## Case presentation

**Figure 1:**
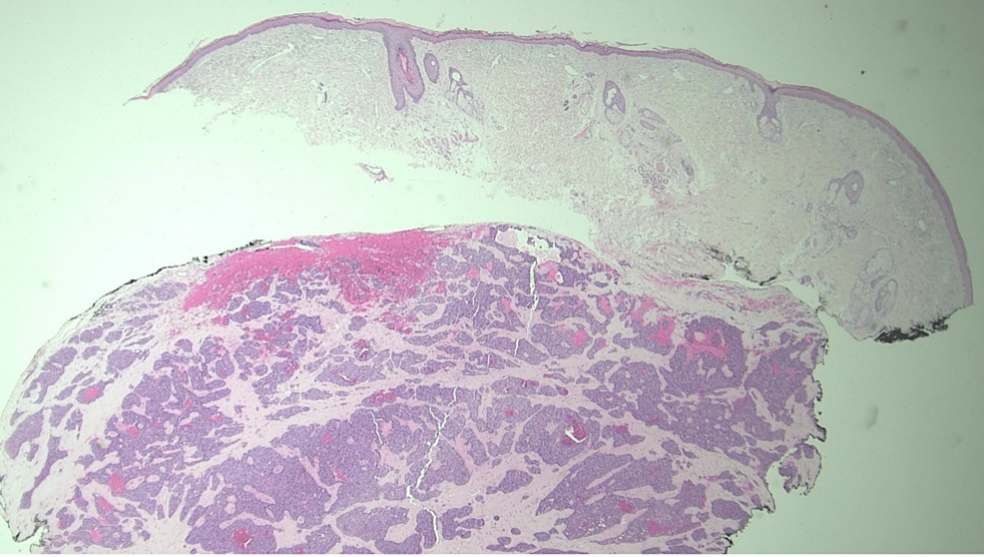
Well-circumscribed neoplasm comprised of basaloid cells arranged in nests with possible focal gland formation (H&E, X20). H&E: hematoxylin and eosin

**Figure 2:**
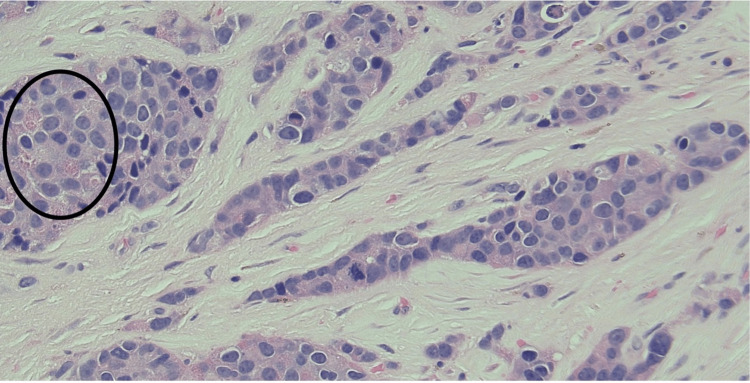
High-power demonstration of cords and nests of malignant cells with scattered zymogen granules (black circle) (H&E, X400).

A 61-year-old white male with a history of a pancreatic mass and a past medical history of rosacea, melanoma, and non-melanoma skin cancers presented to the dermatologic clinic with a painful nodule on his right posterior scalp. A physical exam revealed a 1.2 cm erythematous, tender nodule that was friable and hemorrhagic upon excisional biopsy. Histopathology demonstrated an atypical, dermal-based epithelioid neoplasm, along with focal gland formation and nests containing eosinophilic granules (Figures 1, 2).

**Figure 3:**
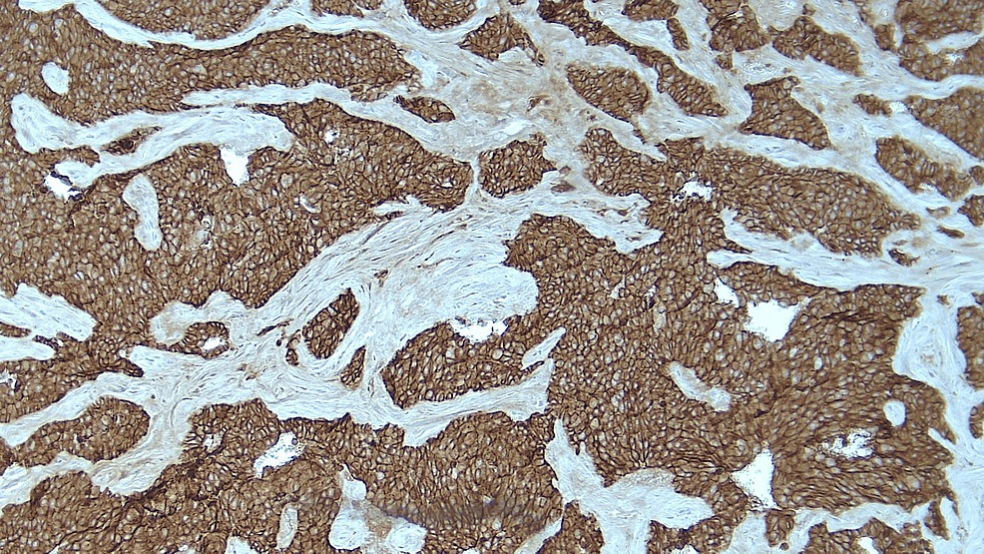
High expression of E-cadherin in PACC (X100).

Immunohistochemistry stains were positive for carcinoembryonic antigen (CEA), cytokeratin (CK) 7, CK 20, synaptophysin, and E-cadherin (Figure 3).

Findings were consistent with PACC based on the clear evidence of zymogen granules, positive stains of interest, and the patient’s history of a primary pancreatic neoplasm. Shortly thereafter, the patient presented with similar complaints of ulcerating lesions on his posterior scalp, confirmed on biopsy as metastatic PACC. At that time, the patient was being treated with chemotherapy for stage III pancreatic cancer.

## Discussion

PACC is a rare, aggressive pancreatic malignancy that typically occurs in white males during the sixth decade of life [3]. Most patients present late in the disease course due to the gradual onset of nonspecific symptoms [3]. Cutaneous metastasis is an uncommon manifestation but is typically discovered in the periumbilical area due to the close proximity of the peritoneum to the epidermis [7,8]. Non-umbilical metastases are notorious for masquerading as various dermatologic conditions, leading to a diverse clinical differential [7,9]. Grossly, metastatic carcinomas appear as painless, nodular, flesh-colored masses or as ulcerative, plaque-like, crusted lesions with an insidious onset [9]. Cutaneous tumors arising from pancreatic cancer typically display a hematogenous spread, leading to the development of tender and erythematous lesions [10]. Microscopically, PACC exhibits glandular architecture and contains highly eosinophilic material due to the presence of zymogen granules [11]. The zymogen granules stain positive for periodic acid-Schiff (PAS) in 95% of all acinar cell carcinomas, which may be sufficient to establish the diagnosis. However, some tissue samples do not contain adequate quantities of zymogen granules, thus requiring additional immunohistochemical staining [11]. Staining for trypsin and chymotrypsin has a high enough sensitivity to detect acinar differentiation [11]. Moreover, immunoreactivity for cytokeratin (CK) 7, CK19, CK20, CEA, and E-cadherin further supports the diagnosis of PACC [11]. High expression of E-cadherin has been strongly associated with malignancies of the pancreas; hence, it may serve as a novel biomarker in pancreatic carcinogenesis [12].

It is important to distinguish between other primary cutaneous scalp neoplasms, such as benign and malignant adnexal tumors. The differential diagnosis of PACC includes primary cutaneous adnexal neoplasms, eccrine adenocarcinomas, sebaceous carcinomas, and apocrine hidradenocarcinomas. Squamous cell carcinoma (SCC), a common non-melanoma skin presentation, is frequently seen by clinicians in the head and neck area as an ulcerative plaque or scaly papule [13]. However, histopathological analysis of SCC reveals prominent keratinization, intercellular bridges, and evident squamous epithelial tissue [13]. Stains of interest for SCC typically include CK 5/6, but its lack of staining for CK 20 differentiates it from the PACC [13]. Additionally, basal cell carcinoma (BCC) is a common neoplastic lesion found on the scalp that appears as a flesh-colored, pearly nodule. BCC displays large nests of basaloid lobules with peripheral palisading on histology [14]. Expression of CKAE1, CKAE3 stains, and BerEP4 markers strongly suggests the diagnosis of BCC [14]. Additionally, eccrine adenocarcinoma should be included in the differential diagnosis because it presents in a similar clinical and histopathological fashion to PACC [15]. Immunohistochemical staining for a sweat gland carcinoma shows a strong reactivity towards the same markers found in PACC, such as CK 7, CK 20, gross cystic disease fluid protein (GCDFP-15), and keratin [15]. However, eccrine adenocarcinoma lacks zymogen granules on microscopical analysis. Furthermore, sebaceous carcinoma (SC) commonly presents in the periocular area and can arise on the scalp as a painless, firm, yellow nodule with associated ulceration [16]. Microscopically, SC is defined by numerous lobules of mature sebocytes and nodular aggregates with reactivity towards androgen receptors, keratin, and adipophilin and a lack of expression of PACC-specific markers [16]. Finally, malignant hidradenocarcinoma should be ruled out because it is derived from apocrine cells, thus staining positive for high-molecular-weight cytokeratin, an epithelial component of the tumor, and CEA [17]. However, hidradenocarcinoma does not express CK7, CK20, or S-100, which would be observed in PACC [17].

When suspecting the diagnosis of a metastatic PACC, prompt evaluation via skin biopsy with immunohistochemistry is paramount, as patients presenting with cutaneous involvement can be the first indication of an underlying malignancy [18]. For instance, in the case reported by Haenen et al., cutaneous lesions were the initial signs of the PACC, which in turn led to an urgent pancreatectomy that prolonged the patient’s life [19]. Early surgical resection of the primary pancreatic neoplasm has been documented to increase the five-year survival rate up to 66%, compared to 17% in unresected patients [20]. Although skin manifestations appear in less than 3% of patients with pancreatic cancer, establishing an early diagnosis of metastatic disease may result in earlier surgical resections and a more favorable prognosis for patients.

## Conclusions

In our case, the definitive diagnosis of PACC was established following the results of the histopathology analysis of the metastatic cutaneous lesion. Generally, pancreatic cancer with widespread metastases has a tremendously low survival rate, and given the aggressiveness and late presentation of these tumors, not all patients qualify for surgical treatment. To conclude, our case highlights the significance of early biopsy and histopathological evaluation in patients with suspicious scalp lesions and a supporting history of illness.
